# Study protocol for a randomised controlled trial to determine the efficacy of lisdexamfetamine for the treatment of acute methamphetamine withdrawal in inpatient settings

**DOI:** 10.1136/bmjopen-2025-107242

**Published:** 2025-11-27

**Authors:** Liam S Acheson, Krista J Siefried, Nicholas Lintzeris, Adrian J Dunlop, Paul S Haber, Shalini Arunogiri, Michael Christmass, Michael Doyle, Mark Donoghoe, Jack Nagle, Brendan Clifford, Rebecca McKetin, Dan I Lubman, Jonathan Brett, Nathan Taylor, Andrew Carr, Frances R Levin, Steven Shoptaw, Nadine Ezard

**Affiliations:** 1 National Centre for Clinical Research on Emerging Drugs, University of New South Wales, Sydney, New South Wales, Australia; 2 Alcohol and Drug Service, St Vincent’s Hospital Sydney, Darlinghurst, New South Wales, Australia; 3 National Drug and Alcohol Research Centre, University of New South Wales, Randwick, New South Wales, Australia; 4 National Centre for Clinical Research on Emerging Drugs, University of New South Wales, Randwick, New South Wales, Australia; 5 Drug and Alcohol Clinical Research and Improvement Network, NSW Health, St Leonards, New South Wales, Australia; 6 South Eastern Sydney Local Health District, Sydney, New South Wales, Australia; 7 Discipline of Addiction Medicine, The University of Sydney, Sydney, New South Wales, Australia; 8 Hunter New England Health, Newcastle, New South Wales, Australia; 9 The University of Newcastle, Newcastle, New South Wales, Australia; 10 Drug Health Services, Sydney Local Health District, Camperdown, New South Wales, Australia; 11 Monash Addiction Research Centre, Eastern Health Clinical School, Faculty of Medicine, Nursing and Health Sciences, Monash University, Melbourne, Victoria, Australia; 12 Turning Point, Eastern Health, Box Hill, Victoria, Australia; 13 Next Step Drug and Alcohol Services, Perth, New South Wales, Australia; 14 National Drug Research Institute, Curtin University, Perth, Western Australia, Australia; 15 Centre for Research Excellence in Aboriginal Health and Alcohol, Discipline of Medicine, Central Clinical School, Faculty of Medicine and Health, The University of Sydney, Sydney, New South Wales, Australia; 16 Clinical Research Unit, UNSW Medicine & Health, Sydney, New South Wales, Australia; 17 The Kirby Institute, University of New South Wales, Sydney, New South Wales, Australia; 18 Real Drug Talk, Melbourne, Victoria, Australia; 19 National Drug and Alcohol Research Centre, University of New South Wales, Sydney, New South Wales, Australia; 20 St. Vincent’s Clinical School, University of New South Wales, Sydney, New South Wales, Australia; 21 School of Population Health, University of New South Wales, Sydney, New South Wales, Australia; 22 Policy, Ethics, and Research, Aboriginal Health & Medical Research Council, Sydney, New South Wales, Australia; 23 Applied Medical Research, St Vincent’s Hospital Sydney, Darlinghurst, New South Wales, Australia; 24 Department of Psychiatry, Columbia University Irving Medical Center, New York State, New York, USA; 25 Division on Substance Use Disorders, New York State Psychiatric Institute, New York, New York, USA; 26 Department of Family Medicine, University of California Los Angeles, Los Angeles, California, USA

**Keywords:** Clinical Trial, Substance misuse, Treatment Outcome, Hospitals

## Abstract

**Introduction:**

Harms due to methamphetamine use disorder (MAUD) are rising globally. Untreated withdrawal symptoms perpetuate the cycle of dependence and are a barrier to treatment. There is no pharmacotherapy approved for methamphetamine withdrawal. Lisdexamfetamine (LDX) dimesylate has potential as an agonist therapy to ameliorate symptom severity during acute methamphetamine withdrawal and increase duration of initial abstinence and retention in treatment.

**Methods and analysis:**

We will conduct a double-blind, randomised, controlled trial to evaluate the efficacy of LDX in reducing symptom severity during acute methamphetamine (MA) withdrawal. One hundred eighty-four adults with moderate to severe MAUD presenting to a health service requesting MA withdrawal treatment who report use of MA within the last 72 hours will be recruited. Participants will be randomised 1:1 to receive a tapering dose of lisdexamfetamine (250 mg on day 1, reducing by 50 mg per day to 50 mg on day 5, followed by 2 days of placebo washout on days 6 and 7), or placebo for 7 days. The study will be conducted over 7 days in an inpatient unit, and all participants will also receive standard inpatient withdrawal care. Participants will be followed up in the community to day 84. The primary outcome is efficacy, defined as the between-group difference in average withdrawal severity measured over the 7-day admission by the Amphetamine Withdrawal Questionnaire. Secondary outcomes are retention in treatment, treatment satisfaction, sleep and concomitant medication use (symptomatic medications and medications for other indications to day 7); safety, craving for MA, post-treatment withdrawal symptoms, depression, anxiety and stress, insomnia and cost effectiveness (to day 28) and MA use, mental, physical and social health and post-withdrawal treatment utilisation (to day 84). A First Nations qualitative substudy will assess the experiences of Aboriginal and Torres Strait Islander participants, ensuring the treatment meets the needs of First Nations people.

**Ethics and dissemination:**

This protocol was first approved by the St Vincent’s Hospital Human Research Ethics Committee on 15/05/2024 (2024/ETH00788). All participants will be provided with a participant information sheet and consent form, be fully informed about the study and given ample time to consider participation. Results will be published in peer-reviewed journals and presented at national and international conferences. Findings will be presented such that individual participants will not be identifiable.

**Trial registration number:**

ACTRN12624001061527.

**STRENGTHS AND LIMITATIONS OF THIS STUDY**

- Sample size powered to detect a meaningful reduction in withdrawal symptoms (the primary outcome) informed by effective treatments for withdrawal from other drugs and published pilot data.
- Uses a high-dose taper of lisdexamfetamine to account for cross-tolerance with methamphetamine. These doses have been shown to be safe in this population.
- Strong community involvement to ensure methods and outcomes meet community needs through consumer investigators, an engaged Consumer Advisory Group and integrated First Nations led substudy.
- Inpatient treatment setting limits generalisability of results and translation to other settings.

## Introduction

 Methamphetamine (MA) is the most widely consumed synthetic stimulant in the world, used by an estimated 30 million people.^1^ The harms associated with MA use have increased over the past 10 years,^2 3^ and this is reflected by increasing demand for treatment.^1 4^ Regular MA use may lead to methamphetamine use disorder (MAUD).^5^ Regular MA use and MAUD are associated with depression, anxiety, psychosis, cardiovascular disease (including ischaemic heart disease, cardiomyopathy and pulmonary arterial hypertension), stroke and increased risk of sexually transmitted or blood-borne viral infections.^6^ MA is now the most common illicit drug for which treatment is sought in Australia, and First Nations people are eight times more likely to seek treatment for MAUD than other Australians.^7^

Poorly managed MA withdrawal is a major obstacle to achieving abstinence from MA, and 88%–97% of people who regularly use MA experience withdrawal.^8 9^ MA withdrawal symptoms include extreme fatigue, poor concentration, cravings, sleep and mood disturbances (including suicidal ideation and attempts^10^) and are of sufficient severity to perpetuate drug use and preclude engagement in treatment for MAUD.^10^,^14^ The acute withdrawal phase starts within 24 hours of last MA use and is most intense in the first 7 days.^14^ MA withdrawal is the first step in the treatment journey, and untreated withdrawal limits capacity to achieve abstinence and sustains the cycle of dependence. Moreover, return to MA use in the context of MA withdrawal establishes negative reinforcement, that is, the removal of negative physical and psychological symptoms during withdrawal by powerful positive response with resumption of MA use.^15^ Withdrawal treatment for substance use disorder of any drug class (eg, nicotine, opioids and cannabis) aims to reduce severity of symptoms^16 17^ and increase completion of withdrawal and engagement with ongoing treatment in the post-withdrawal period.^18^

There is no effective treatment for MA withdrawal.^19^ Current clinical practice is marked by poor outcomes, inconsistent approaches and a limited evidence base.^20^ Symptomatic medications (eg, short-term benzodiazepines) may reduce agitation and irritability but do not effectively manage the broader range of MA withdrawal symptoms (ie, craving, poor concentration and fatigue) and risk clinically significant side effects.^20^ Medications with similar modes of action to the drug being stopped have been shown to reduce withdrawal severity and are effective in treating other withdrawal syndromes (eg, nicotine for tobacco withdrawal, buprenorphine for opioids and nabiximols for cannabis).^16 17 21^ Dexamphetamine and other stimulants can reduce withdrawal symptoms in clinical trials for MAUD; however, there are no randomised controlled studies directed specifically at using these medications to manage MA withdrawal.^19^

Lisdexamfetamine (LDX) is a candidate agonist pharmacotherapy for MA withdrawal. It is a pharmacologically inactive prodrug of dexamphetamine that increases extracellular dopamine.^22 23^ LDX (to a maximum dose of 70 mg/day) is approved in Australia for the treatment of attention deficit hyperactivity disorder (ADHD) and binge eating disorder.^24^ When compared with immediate release dexamphetamine, LDX results in slower onset of drug effects and lower peak concentration of dopamine (maximum dexamphetamine concentrations achieved 3.5 hours after dosing, duration of clinical action 10–12 hours), allowing for once-daily administration.^25^ LDX offers potential advantages for the treatment of MA withdrawal in the community due to lower potential for non-medical use as intravenous LDX does not result in more rapid onset of action than oral administration and the blunted dopaminergic action may result in reduced positive mesolimbic reinforcement.^26^ Our group recently completed an open-label, single-arm pilot study that examined a 5-day tapering regimen of LDX starting at 250 mg and decreasing by 50 mg a day among adults in acute MA withdrawal over a 7-day inpatient period (n=10). Results demonstrated safety and feasibility of the proposed randomised controlled trial (RCT) methods and dosing regimen.^27^ In qualitative interviews, participants of the pilot trial reported that the intervention was highly acceptable and thought that LDX helped create an easier withdrawal experience.^28^

Early MA abstinence and successful MA withdrawal are powerful predictors of longer term treatment outcomes.^29^ As the first step towards reducing use or achieving abstinence, lack of effective withdrawal treatment delays engagement with healthcare services.^30^ Failure to complete a withdrawal attempt from MA is a major barrier to retention in subsequent treatment, while early treatment engagement is associated with better substance use outcomes.

Pilot data demonstrated promising preliminary efficacy signals for LDX’s ability to modulate withdrawal symptoms during the acute withdrawal period. Further, the safety profile of LDX, demonstrated by previous studies at doses up to 250 mg once daily in people with MAUD, is encouraging and in line with the published product label.^27 31^ LDX therefore has potential as a future treatment option for MA withdrawal.

### Objectives

The objective of this study is to assess the efficacy of a tapering dose regimen of LDX to ameliorate the symptoms of acute MA withdrawal over 7 days.

We hypothesise that treatment with oral LDX will result in significantly reduced MA withdrawal symptoms when compared with placebo, as measured daily by the validated Amphetamine Withdrawal Questionnaire (AWQ) over a 7-day period. The primary objective of this trial is to determine the efficacy of a tapering dose regimen of LDX, starting at 250 mg once daily over 5 days followed by a 2-day wash-out period, in reducing MA withdrawal symptom severity.

Secondary objectives examine differences between the two randomised groups across the following:

- Withdrawal episode (to day 7): including retention in inpatient withdrawal treatment episode, treatment satisfaction and self-reported effectiveness, sleep, concomitant medication utilisation and quality of blinding over 7 days.
- Immediate post-withdrawal period (to day 28): safety, craving for MA, post-treatment withdrawal symptoms, depression, anxiety and stress, insomnia and cost-effectiveness over 28 days.
- Extended post-withdrawal period (to day 84): MA use, mental, physical and social health and post-withdrawal treatment utilisation over 84 days.

## Methods and analysis

### Trial design

We will conduct a multisite, two-arm, placebo-controlled, randomised trial. The primary endpoint of this study is the average daily AWQ score while on treatment (up to day 7). Secondary endpoints will also be measured up to days 7, 28 and 84. A Standard Protocol Items: Recommendations for Interventional Trials checklist is included as supplementary material. Participants will be recruited between June 2025 and September 2027. This study is conceptualised as a phase 3 clinical trial but is technically phase 4 given lisdexamphetamine’s post-marketing status.

### Sample size

A sample size of 128 participants who receive at least one dose of study medication is required to detect a moderate effect size (d=0.5) in the between-group difference for mean AWQ scores with 80% power at a significance level 0.05. This effect size corresponds to a difference of 2.5 in mean AWQ scores over the 7-day withdrawal period, based on our pilot study data (SD 5.1^27^) and is consistent with clinically meaningful changes in withdrawal scales for cannabis and opioid withdrawal trials.^21 32^ Assuming 30% attrition, a sample size of 184 randomised participants (92 in each group) is required to maintain sufficient power. An estimated 20% of the study population is expected to be eligible for the First Nations substudy (based on national treatment data^7^).

### Participants

This study will be conducted in five Australian sites with inpatient withdrawal management units experienced in delivering and evaluating interventions for MAUD. The participating sites are located in New South Wales, Victoria and Western Australia.

The study population will comprise adults presenting to inpatient drug treatment services seeking MA withdrawal treatment, determined to have moderate to severe MAUD by an Addiction Medicine Specialist or Psychiatrist (Diagnostic and Statistical Manual of Mental Disorders 5^th^ Edition, Text Revision (DSM-5-TR) criteria^5^), reporting last use of MA within 72 hours of first dose of study drug, providing a point-of-care urine drug screen positive for MA and who provide written informed consent.

Participants will be excluded if they present with moderate to severe use disorder of alcohol, opioids and/or sedative hypnotics or anxiolytics (benzodiazepines or gamma-hydroxybutyrate) (based on DSM-5-TR criteria); are lactating, pregnant or of childbearing potential and not willing to avoid becoming pregnant during the treatment phase of the study; have acute severe mental or physical comorbidity that would interfere with study participation as assessed by the site principal investigator (PI); are currently experiencing active psychosis (defined by a score of 4 or greater on at least two items of the psychosis (suspiciousness, hallucinations and unusual thought content) items of the Brief Psychiatric Rating Scale or current active suicidality (defined by a high risk (by answering yes to questions 4, 5 or 6 within the past 3 months) on the Columbia Suicide Severity Rating Scale Screener^33^); have exposure to LDX, dexamphetamine, modafinil or methylphenidate in the 4 weeks prior to screening; have contraindications to LDX (per product label, eg, advanced arteriosclerosis, symptomatic cardiovascular disease including cardiac arrhythmia, ischaemic heart disease, moderate to severe hypertension (systolic blood pressure≥160 mm Hg and/or diastolic blood pressure (BP) ≥100 mm Hg), excepting drug dependence^24^) or are currently enrolled in another study that would interfere with study participation.

Potential participants will be recruited directly from those presenting to the services and through external advertising (posters, flyers, social and traditional media), which will provide a link for people to complete an expression of interest form. Those interested will be prescreened in person or by telephone by a researcher either on presentation to the scheduled admission to the ward or prior. Potentially eligible participants will be invited to provide informed consent and proceed with a formal eligibility assessment by a specialist in addiction medicine or psychiatry on admission.

### Randomisation

The randomisation schedule will be developed in partnership with the study biostatistician and will be implemented in Research Electronic Data Capture (REDCap).^34 35^ Participants will be randomised in a 1:1 ratio to either receive a tapering dose of LDX or matched placebo, using variable block randomisation (to help maintain allocation concealment), stratified by site. Participants will be randomised by site clinical trial pharmacists, who have no participant contact.

Treatment allocation in this trial is double-blinded. Study medication and placebo will be compounded to be indistinguishable by an independent clinical trial pharmaceutical service and packaged centrally to account for tapering dose and maintaining blind. Site pharmacists will dispense labelled study drug with the participant’s identification number and site. Aside from site trial pharmacists and an unblinded statistician (who have no participant contact), to maintain the double-blind, no member of the study team or clinical staff will know the treatment allocation (active/placebo).

### Outcomes and measures

The primary outcome of this study is efficacy, defined as the between-group mean difference in average withdrawal severity while on treatment, measured daily using the AWQ. The AWQ is a structured questionnaire measuring severity of amphetamine withdrawal symptoms including cravings, mood, anhedonia, anxiety, agitation, energy or blunted movements, sleep and disturbing dreams.^36^ It is the only validated measure of amphetamine withdrawal and has demonstrated internal validity for MA withdrawal.

Secondary outcomes assessed over 7 days are between-group differences in retention in treatment; treatment satisfaction and perceived medication effectiveness measured by 100 mm visual analogue scale (VAS); sleep measured by continuous actigraphy, daily sleep diaries (Consensus Sleep Diary^37^) and Insomnia Severity Index (ISI)^38^; concomitant medication use (symptomatic medications—anxiolytics and sedatives and medications for other indications) and quality of the blind assessed using a simple blinding index.^39^ Safety will be assessed by adverse events (AEs) (number, seriousness, severity, causality) over 28 days. Other 28-day outcomes are between-group differences in days of self-reported MA use over the last 28 days measured by the Timeline Followback (TLFB) method^40^; craving for MA measured by a 100 mm VAS; post-treatment withdrawal symptoms measured by AWQ; depression, anxiety and stress measured by the Depression, Anxiety and Stress Scale 21^41^ and insomnia measured by the ISI all assessed weekly to day 28. Cost-effectiveness will be assessed using an incremental cost-effectiveness ratio (ICER). Outcomes assessed over an 84-day period will be between-group differences in days of self-reported MA use over the last 28 days measured by the TLFB^39^; mental, physical and social health measured by the Patient Reported Outcomes Measurement Information System 29 (PROMIS-29)^42^ and self-reported post-withdrawal treatment utilisation assessed every 4 weeks to day 84. The full schedule of assessments is available in table 1.

**Table 1:** Schedule of assessments

Assessment/procedure		Study day										
		Sc/0*	1	2	3	4	5	6	7	SFU†	LFU‡	Wd§
*Screening, eligibility*	Consent	●										
	Human chorionic gonadotropin (hCG) urine	●										
	Point-of-care urine drug test	●										
	Demographics	●										
	DSM-5 checklist¶^5^	●										
	Medical assessment	●										
	Brief Psychiatric Rating Scale (BPRS) (suspiciousness, hallucination and unusual thought content subscales)	●										
	Columbia Suicide Severity Rating Scale (C-SSRS) Screener^33^	●										
*Study procedures*	Randomisation**	●										
	Study drug (active or placebo)		●	●	●	●	●	●	●			
*Primary outcome*	Withdrawal: Amphetamine Withdrawal Questionnaire (AWQ)^36^	●	●	●	●	●	●	●	●			
*Secondary outcomes*	*Safety*											
	Adverse events	●	●	●	●	●	●	●	●	●		●
	Vital signs (as available at baseline and four times daily thereafter)	●	●	●	●	●	●	●	●			
	*Questionnaires—physical, emotional health*											
	Post-treatment withdrawal: AWQ									●		●
	MA craving: visual analogue scale	●	●	●	●	●	●	●	●	●		●
	Substance use: Timeline Follow-back††^39^	●							●	●	●	●
	Depression, anxiety: DASS-21^41^	●							●	●		●
	Physical, mental, social health: Patient-Reported Outcomes Measurement Information System (PROMIS-29)^42^	●							●	●	●	●
	Treatment satisfaction and perceived effectiveness, additional question on perceived allocation ‡‡								‡‡			
	*Sleep*											
	Continuous actigraphy§§	●	●	●	●	●	●	●	●			●
	Consensus sleep diary^37^		●	●	●	●	●	●	●			●
	Insomnia: Insomnia Severity Index (ISI)^38^	●							●	●		●
	*Other*											
	Qualitative interview (substudy participants only)¶¶								●	●		
	Concomitant medications and psychosocial care	●	●	●	●	●	●	●	●	●	●	●
	Health service utilisation								●	●	●	●
	Retention in treatment: doses of study drug, adherence; withdrawal treatment completion		●	●	●	●	●	●	●			
	Methamphetamine use history	●										
*Other*	Substance use treatment history	●										
	Substance Use Recovery Goals and Expectations: SURGE	●										
	Childhood ADHD screen: Wender Utah Rating Scale (WURS)^49^	●										
	Participant reimbursement	●							●	●	●	

* Day 0/baseline (screening and baseline).

† Short-term follow-up at days 14, 21 and 28.

‡ Long-term follow-up at days 56 and 84.

§ Withdrawal (refer to Section 11 of the protocol for details).

¶ For methamphetamine, alcohol, benzodiazepines, gamma-hydroxybutyrate (GHB) and opioids.

** May be conducted early on day 1 if required, day 1 medication administration before 10am.

†† Past 28-day Timeline Followback (TLFB) to be conducted at baseline to include methamphetamine (MA) and other substances, subsequent administrations to assess MA only and cover time period since last scheduled visit.

‡‡ Treatment satisfaction and perceived efficacy are to be collected at the early discontinuation visit during the inpatient period D0–D7. It is not required if the discontinuation/withdrawal is post-discharge.

§§ Wearable sleep monitoring device: Geneactive.

¶¶ Interviews will be at day 7 (±4 days) and between days 21 and 28.

ADHD, attention deficit hyperactivity disorder; AWQ, Amphetamine Withdrawal Questionnaire; DASS-21, Depression, Anxiety and Stress Scale 21; DSM-5, Diagnostic and Statistical Manual of Mental Disorders 5th Edition; MA, methamphetamine.

AEs will be classified according to the preferred term and system organ class of the Medical Dictionary for Regulatory Activities (V.27.1) and evaluated for seriousness, severity and relatedness to study medication by site PIs, who will be blinded to group allocation. Serious adverse events (SAEs) will be evaluated for relatedness by the Trial Chairperson (coordinating PI) who will also be blinded to group allocation. All AE data (and their group allocation) will be reviewed by the independent data safety monitoring board (DSMB).

### Intervention

The clinical intervention is designed as a 7-day inpatient treatment programme. Each participant will be offered standard of care withdrawal management services available at the site (eg, concomitant medications such as diazepam and psychosocial support such as counselling), all other wraparound services (eg, accommodation and meals) and linkages with post-withdrawal care and ongoing treatment and support (eg, counselling and residential rehabilitation). Post-withdrawal care will be dictated by the patient’s own goals and discharge plan. In addition, participants will receive daily supervised administration of study medication (5 days of reducing LDX plus 2 days of placebo washout, or 7 days of placebo). The placebo is prepared in matching gelatine capsules filled with microcrystalline cellulose. Participants may refuse to take the study medication if they wish.

### Study procedures

Day 0 is defined as the day of admission to the unit. Participants provide written informed consent, are formally screened for eligibility and enrolled in the trial at this time. Participants will then be randomised and baseline data collected, including presence of childhood ADHD symptomatology. Participants will undergo a medical review prior to enrolment and at discharge from the unit.

Participants will receive a tapering dose of LDX, beginning at 250 mg on day 1, reducing by 50 mg per day until 50 mg on day 5, delivered via 50 mg capsules (table 2). This dose was chosen as 250 mg once daily was found to be safe and tolerable in people with MAUD for up to 12 weeks,^27 31 43^ rapid tapering LDX for MA withdrawal was acceptable to people with MAUD^28^ and the high initial dose accounts for cross-tolerance with MA. A 2-day washout period is required to assess possible rebound withdrawal symptoms occurring after discontinuation of LDX, which may be expected considering similar experiences with agonist treatments for opioid withdrawal and an effect noted in the pilot study, leading to a 7-day treatment protocol. The dosing taper will also be blinded to participants: all participants will receive five capsules per day. In the placebo arm, this will be five placebo capsules per day. In the treatment arm, this will be five active capsules on day 1, reducing by one active capsule per day to five placebo capsules on days 6 and 7 (table 3). Each day, participants will complete outcome assessments and a medical review as per the schedule of assessments. Participants will have their vital signs recorded four times a day, and excursions outside predefined limits will be considered an AE (systolic blood pressure≥180 mm Hg or <100 mm Hg, heart rate≥120 bpm or temperature≥38.5°C or <35.5°C). If a participant experiences a grade 3 or grade 4 AE that is considered to be related to the study medication, no further study medication will be dispensed to the participant until the AE is resolved.

**Table 2:** Study group allocation

		Inpatient withdrawal							Post-discharge follow-up in the community				
Investigational product		Day 1	Day 2	Day 3	Day 4	Day 5	Day 6	Day 7	Day 14	Day 21	Day 28	Day 56	Day 84
1:1 ratio	LDX OD*	250 mg	200 mg	150 mg	100 mg	50 mg	0 mg	0 mg	*nil*	*nil*	*nil*	*nil*	*nil*
	Placebo OD*	0 mg	0 mg	0 mg	0 mg	0 mg	0 mg	0 mg	*nil*	*nil*	*nil*	*nil*	*nil*

* OD, daily at 09:00±1 hour.

LDX, lisdexamfetamine.

**Table 3:** Active and placebo capsule allocation

		Inpatient withdrawal						
Investigational product		Day 1	Day 2	Day 3	Day 4	Day 5	Day 6	Day 7
1:1 ratio	LDX OD	5 active	4 active 1 placebo	3 active 2 placebo	2 active 3 placebo	1 active 4 placebo	5 placebo	5 placebo
	Placebo OD	5 placebo	5 placebo	5 placebo	5 placebo	5 placebo	5 placebo	5 placebo

* OD, daily at 09:00±1 hour.

LDX, lisdexamfetamine.

Participants will be discharged from hospital on day 7, and no further intervention will be available as part of the study; however, participants will be able to engage on ongoing substance use treatment thereafter. Participants will be followed up in the community on days 14, 21, 28, 56 and 84 post-baseline via telephone, in person or self-completed survey delivered via short message service or email.

A First Nations qualitative substudy will be conducted, which aims to explore the experiences of Aboriginal and Torres Strait Islander participants. The medication trialled should have similar physiological effects for Aboriginal and non-Aboriginal people, but we hypothesise that sociocultural contexts may affect the treatment experience. This substudy will be led by First Nations investigators (MD and NT). All First Nations participants will be invited to participate in two qualitative interviews, anticipated to be 30–60 min each. A First Nations research officer will support the substudy and conduct the interviews, remotely on discharge from hospital and between days 21 and 28 (via telephone, video conferencing or face-to-face). Interviews will be semistructured and use a research yarning methodology, a semistructured interview technique involving informal and relaxed discussion through which both the researcher and participant share experiences that are related to places and topics of interest relevant to the research study. These experiences are related as stories, or yarns. While the yarns in this study are relaxed and interactive, they are purposeful with a defined beginning and end in order to obtain a clear understanding of the participants’ experience of MA withdrawal and the study medication. This is distinct from social or collaborative yarning.^44^ Yarning is a process that is centred around relationship building and requires the researchers to be accountable to Indigenous people participating in the research, demanding human-to-human interaction.^45^

LDX will not be available for the management of MAUD post-discharge; however, standard care ongoing treatment individualised to the participant, including but not limited to ongoing counselling or referral to residential rehabilitation services, will be provided.

Participants who consent to partake in the study and complete all the screening and baseline assessments will be reimbursed with $20. If a participant remains admitted to the ward until the primary endpoint (day 7), they will receive an additional $30. Participants will receive $40 for attending on day 14, $50 for attending on day 21, $60 for attending on day 28 and $40 for each of the day 56 and 84 follow-up visits. The maximum total reimbursement is $280. An additional $60 reimbursement will be provided per qualitative interview completed.

Participants who choose to discontinue the per-protocol treatment will be offered usual treatment as per unit protocol and complete all remaining research assessments and interviews. For those who revoke consent, no further information will be collected. However, data collected up to their discontinuation from the study will be retained.

The cost-effectiveness of this intervention will also be evaluated.

### Statistical methods

*Statistics:* All outcome variables will be analysed under intention-to-treat principles, including all participants who received at least one dose of the study medication, with missing data handled using multiple imputation.^46^ A linear regression model will be used to analyse the primary efficacy measure of the between-group difference in average on-treatment AWQ scores, adjusting for site and baseline score, with robust standard errors accounting for potential heteroskedasticity. Adherence to study drug will be summarised to day 5 (medication dosing is administered and observed by clinical staff), and ordinal logistic regression will be used to compare the retention of participants in treatment (to day 7) between study arms. Secondary endpoints will be analysed using regression models as appropriate for the type of outcome, with likelihood-based mixed effects models used for repeated measures. For discrete outcomes such as the incidence of AEs, differences in rates between groups will be analysed using generalised linear models. The quality of the blind will be tested using a simple blinding index for randomised controlled trials.^39^ All tests of treatment effect will be conducted at a two-sided significance level of 0.05.

*First Nations led substudy:* Thematic framework analysis will assess emerging themes in the data, analysed through a cultural lens overseen by the Aboriginal Reference Group. No a priori theories will be applied to the data. Qualitative analysis of the interviews will be conducted under the supervision of First Nations Investigators.

*Health economics:* A cost-effectiveness evaluation will be conducted alongside the RCT, from a health system perspective, to assess the cost:outcomes ratio. Resource use will include all clinical services provided as trial interventions, management of SAEs and self-reported health service utilisation post-discharge from hospital (including general practitioner, nurse practitioner, emergency department, hospital admission, specialist doctor, allied health or any other health service use). Societal costs (ie, justice) will not be included in the analysis. Consistent with other reported economic evaluations of drug treatments, the primary economic outcome will be quality-adjusted life years measured by the PROMIS-29, self-reported engagement in treatment and health service utilisation (days 7, 14, 21, 28, 56 and 84). The ICER will be calculated as (C_LDX_-C_Control_)/(E_LDX_-E_Control_). Uncertainty analysis will be conducted using non-parametric bootstrapping to generate intervals around the ICER. Sensitivity analysis will assess the effect of variation in key variables.

### Data safety monitoring board

An independent DSMB will be established prior to study recruitment, and the DSMB membership will include an addiction medicine specialist or addiction psychiatrist, a clinical trialist and a biostatistician (all not otherwise involved with the study). The DSMB will convene following the first participant, first visit and quarterly thereafter. All SAEs, suspected unexpected serious adverse reactions and pooled AWQ scores (primary efficacy outcome) will be reviewed by the DSMB every 6 months. Following each meeting, the DSMB will advise one of four options: continue study as per protocol, continue study with protocol amendments, suspend study or discontinue study.

### Data statement

This study will use electronic data capture in the form of REDCap.^35^ The REDCap database is located on a standalone database server hosted by the Hunter Medical Research Institute (HMRI). The database server resides behind the HMRI internal firewall, and access to the server is controlled via firewall rules. All data collected via REDCap are backed up daily, both on the local server and by the HMRI backup system. All connections to the system, both external and internal, will occur over encrypted channels.

The exception to this is for interview data provided during the First Nations substudy. To ensure First Nations ownership of data and control of research processes, the First Nations researcher conducting the interviews through the University of Sydney will collect audio recordings of the interviews and keep them on a password-protected server located at the University of Sydney. Only First Nations researchers directly involved in the collection of interviews will have access to these data.

### Ethics and dissemination

This protocol was first approved by the St Vincent’s Hospital Human Research Ethics Committee (HREC) on 15/05/2024 (2024/ETH00788). This protocol received additional approval from the Aboriginal Health and Medical Research Council of New South Wales (NSW) HREC on 09/10/2024 and was prospectively registered on the Australian New Zealand Clinical Trials Registry on 03/09/2024 (ACTRN12624001061527).

All participants will be provided with a participant information sheet and consent form, be fully informed about the study and given ample time to consider participation prior to study entry. If agreeable to participate, the participant will be asked to sign a consent form. Any participant who identifies as Aboriginal and/or Torres Strait Islander will also be provided information about the First Nations substudy and invited to participate and, if interested, will be given a separate information and consent form for the substudy. The decision to participate in the substudy will not affect participation in the parent study.

To ensure anonymity, each participant will be allocated a unique identifier at randomisation, and no identifiable information will be stored with participant study data. The results of this study will be published in peer-reviewed journals and presented at national and international conferences. In all publications, results will be presented such that individual participants will not be identifiable in any way.

### Patient and public involvement statement

The initial concept of investigating the use of LDX in reducing MA use in people who are dependent on MA was proposed by a client who prefers to be unnamed. This protocol was developed with an investigator who has lived experience of substance use and is overseen by a Consumer Advisory Group, consisting of people with lived or living experience. This protocol was also developed by First Nations investigators and a First Nations Advisory Group, which includes Aboriginal and Torres Strait Islander people. These groups will oversee the implementation of the protocol and ensure that the results of the study are interpreted with the perspectives of the communities most affected by this research. Further, participants in the pilot study were asked to reflect on receiving this treatment programme,^28^ and their experiences informed the development of this protocol.

## Discussion

This study protocol is for the first randomised, controlled trial assessing the efficacy of LDX for acute MA withdrawal. The strength of this study is its proposed sample size, which is powered to detect a meaningful reduction in withdrawal symptoms (the primary outcome), informed by effective treatments for withdrawal from other drugs and published pilot data. The study uses doses of LDX starting over three times higher than the maximum dose for ADHD to account for cross-tolerance between MA and other amphetamines, with a rapid taper during the period when withdrawal symptoms are at their most intense.^14^ The inpatient design of this trial will ensure close monitoring of participants and 24-hour nursing and medical support, as well as a safe location allowing for people with social conditions (eg, housing) to participate. A strong consumer voice in the development of this protocol through lived/living experience investigators and a Consumer Advisory Group, as well as an integrated First Nations-led substudy, is also a strength. This protocol has limitations, including the fixed-dose design, limited duration of active treatment (reflecting typical duration in public hospital inpatient facilities in Australia) and requirement for participants to have access to, and be admitted to, a hospital-based withdrawal management unit, potentially limiting generalisability of results to other settings and restricting the ability of people with caring responsibilities, restrictive employment or prior traumatic experiences in similar settings to participate. Despite these barriers, however, 4170 people in Australia accessed withdrawal management for amphetamines in 2022–2023, the second most common drug of concern for withdrawal management behind alcohol.^47^ Importantly, agonist treatment approaches are common in Australian treatment settings for other drugs of concern,^48^ and a similar approach for treating MA withdrawal could be implemented within existing treatment structures. The primary outcome chosen for this study is a reduction in withdrawal symptoms as measured by the AWQ, reflective of the aims of withdrawal treatment to safely and comfortably complete a withdrawal episode,^48^ and aligns with studies in opioid withdrawal treatment.^21^ The long follow-up and secondary outcomes will assess MA use and linkages with treatment services and provide much needed data on clinically meaningful reductions in substance use and longer term treatment outcomes post-withdrawal. If effective in this setting, outpatient studies to determine the safety and efficacy of LDX to manage ambulatory MA withdrawal and expansion to people with other co-existing conditions or substance use disorders are warranted. If effective, this intervention will provide the first pharmacological treatment option for acute MA withdrawal, helping people reduce their MA use and allowing them to meet their treatment goals.

## Supplementary material

10.1136/bmjopen-2025-107242 online supplemental file 1

## References

[1] United Nations Office on Drugs and Crime (2024). World drug report 2024.

[2] Jones CM, Houry D, Han B (2022). Methamphetamine use in the United States: epidemiological update and implications for prevention, treatment, and harm reduction. Ann N Y Acad Sci.

[3] McKetin R, Degenhardt L, Shanahan M (2018). Health service utilisation attributable to methamphetamine use in Australia: Patterns, predictors and national impact. Drug Alcohol Rev.

[4] Man N, Sisson SA, McKetin R (2022). Trends in methamphetamine use, markets and harms in Australia, 2003-2019. Drug Alcohol Rev.

[5] (2013). Diagnostic and Statistical Manual of Mental Disorders: DSM-5.

[6] Karila L, Petit A, Cottencin O (2010). Methamphetamine dependence: Consequences and complications. *Presse Med*.

[7] Australian Institute of Health and Welfare (2023). Alcohol and other drug treatment services in Australia annual report.

[8] Cantwell B, McBride AJ (1998). Self detoxication by amphetamine dependent patients: a pilot study. Drug Alcohol Depend.

[9] Schuckit MA, Daeppen JB, Danko GP (1999). Clinical implications for four drugs of the DSM-IV distinction between substance dependence with and without a physiological component. Am J Psychiatry.

[10] Meredith CW, Jaffe C, Ang-Lee K (2005). Implications of chronic methamphetamine use: a literature review. Harv Rev Psychiatry.

[11] Brecht M-L, von Mayrhauser C, Anglin MD (2000). Predictors of Relapse After Treatment for Methamphetamine Use. J Psychoactive Drugs.

[12] Galloway GP, Singleton EG, Anglin MD (2008). How Long Does Craving Predict Use of Methamphetamine? Assessment of Use One to Seven Weeks after the Assessment of Craving. Subst Abuse.

[13] Hartz DT, Frederick-Osborne SL, Galloway GP (2001). Craving predicts use during treatment for methamphetamine dependence: a prospective, repeated-measures, within-subject analysis. Drug Alcohol Depend.

[14] McGregor C, Srisurapanont M, Jittiwutikarn J (2005). The nature, time course and severity of methamphetamine withdrawal. Addiction.

[15] Song S-H, Kim S, Jang W-J (2024). Exploring the progression of drug dependence in a methamphetamine self-administration rat model through targeted and non-targeted metabolomics analyses. Sci Rep.

[16] Werneck MA, Kortas GT, de Andrade AG (2018). A Systematic Review of the Efficacy of Cannabinoid Agonist Replacement Therapy for Cannabis Withdrawal Symptoms. CNS Drugs.

[17] Allsop DJ, Copeland J, Lintzeris N (2014). Nabiximols as an agonist replacement therapy during cannabis withdrawal: a randomized clinical trial. JAMA Psychiatry.

[18] Timko C, Below M, Schultz NR (2015). Patient and program factors that bridge the detoxification-treatment gap: a structured evidence review. J Subst Abuse Treat.

[19] Acheson LS, Williams BH, Farrell M (2023). Pharmacological treatment for methamphetamine withdrawal: A systematic review and meta-analysis of randomised controlled trials. Drug Alcohol Rev.

[20] Lintzeris N, Sunjic S, Demirkol A (2019). Management of withdrawal from alcohol and other drugs: an evidence check rapid review brokered by the sax institute (www.saxinstitute.org.au) for the nsw ministry of health.

[21] Gowing L, Ali R, White JM Buprenorphine for managing opioid withdrawal. Cochrane Database of Systematic Reviews.

[22] Sharman J, Pennick M (2014). Lisdexamfetamine prodrug activation by peptidase-mediated hydrolysis in the cytosol of red blood cells. Neuropsychiatr Dis Treat.

[23] Pennick M (2010). Absorption of lisdexamfetamine dimesylate and its enzymatic conversion to d-amphetamine. Neuropsychiatr Dis Treat.

[24] (2013). Australian product information: vyvanse (lisdexamfetamine dimesilate). https://www.ebs.tga.gov.au/ebs/picmi/picmirepository.nsf/PICMI?OpenForm&t=&q=lisdexamfetamine.

[25] Krishnan SM, Pennick M, Stark JG (2008). Metabolism, Distribution and Elimination of Lisdexamfetamine Dimesylate. Clin Drug Investig.

[26] Jasinski DR, Krishnan S (2009). Abuse liability and safety of oral lisdexamfetamine dimesylate in individuals with a history of stimulant abuse. *J Psychopharmacol*.

[27] Acheson LS, Ezard N, Lintzeris N (2022). Lisdexamfetamine for the treatment of acute methamphetamine withdrawal: A pilot feasibility and safety trial. Drug Alcohol Depend.

[28] Acheson LS, Clay S, McKetin R (2024). Participant experiences in a pilot study for methamphetamine withdrawal treatment: Implications for retention. Int J Drug Policy.

[29] Brensilver M, Heinzerling KG, Swanson AN (2012). Placebo-group responders in methamphetamine pharmacotherapy trials: the role of immediate establishment of abstinence. Exp Clin Psychopharmacol.

[30] Cumming C, Troeung L, Young JT (2016). Barriers to accessing methamphetamine treatment: A systematic review and meta-analysis. Drug Alcohol Depend.

[31] Ezard N, Clifford B, Dunlop A (2021). Safety and tolerability of oral lisdexamfetamine in adults with methamphetamine dependence: a phase-2 dose-escalation study. BMJ Open.

[32] Brezing CA, Levin FR (2018). The Current State of Pharmacological Treatments for Cannabis Use Disorder and Withdrawal. *Neuropsychopharmacol*.

[33] Posner K, Brown GK, Stanley B (2011). The Columbia-Suicide Severity Rating Scale: initial validity and internal consistency findings from three multisite studies with adolescents and adults. Am J Psychiatry.

[34] Harris PA, Taylor R, Minor BL (2019). The REDCap consortium: Building an international community of software platform partners. J Biomed Inform.

[35] Harris PA, Taylor R, Thielke R (2009). Research electronic data capture (REDCap)--a metadata-driven methodology and workflow process for providing translational research informatics support. J Biomed Inform.

[36] Srisurapanont M, Jarusuraisin N, Jittiwutikan J (1999). Amphetamine Withdrawal: I. Reliability, Validity and Factor Structure of a Measure. *Aust N Z J Psychiatry*.

[37] Carney CE, Buysse DJ, Ancoli-Israel S (2012). The consensus sleep diary: standardizing prospective sleep self-monitoring. Sleep.

[38] Bastien CH, Vallières A, Morin CM (2001). Validation of the Insomnia Severity Index as an outcome measure for insomnia research. Sleep Med.

[39] Petroff D, Bacak M, Dagres N (2024). A simple blinding index for randomized controlled trials. Contemp Clin Trials Commun.

[40] Robinson SM, Sobell LC, Sobell MB (2014). Reliability of the Timeline Followback for cocaine, cannabis, and cigarette use. Psychol Addict Behav.

[41] Sinclair SJ, Siefert CJ, Slavin-Mulford JM (2012). Psychometric evaluation and normative data for the depression, anxiety, and stress scales-21 (DASS-21) in a nonclinical sample of U.S. adults. Eval Health Prof.

[42] Cook KF, Jensen SE, Schalet BD (2016). PROMIS measures of pain, fatigue, negative affect, physical function, and social function demonstrated clinical validity across a range of chronic conditions. J Clin Epidemiol.

[43] Ezard N, Clifford B, Siefried KJ (2025). Lisdexamfetamine in the treatment of methamphetamine dependence: A randomised, placebo-controlled trial. Addiction.

[44] Bessarab D, Ng’andu B (2010). Yarning About Yarning as a Legitimate Method in Indigenous Research. *IJCIS*.

[45] Spradley JP (2016). The Ethnographic Interview.

[46] Rubin DB (2004). Multiple Imputation for Nonresponse in Surveys.

[47] Australian Institute of Health and Welfare (2024). Alcohol and other drug treatment services in australia annual report.

[48] NSW Ministry of Health (2022). Management of withdrawal from alcohol and other drugs: clinical guidance.

[49] Ward MF, Wender PH, Reimherr FW (1993). The Wender Utah Rating Scale: an aid in the retrospective diagnosis of childhood attention deficit hyperactivity disorder. Am J Psychiatry.

