# Supplementary material for: Study protocol for a randomised controlled trial to determine the efficacy of lisdexamfetamine for the treatment of acute methamphetamine withdrawal in inpatient settings
Source: BMJ Open. 2025 Nov 27;15(11):e107242. doi: 10.1136/bmjopen-2025-107242 (PMC12684119; doi:10.1136/bmjopen-2025-107242)
Supplement: Supplementary file 2 [file bmjopen-2025-107242.reviewer_comments.pdf]

PEER REVIEW HISTORY

BMJ Open publishes all reviews undertaken for accepted manuscripts. Reviewers are asked to complete a checklist review form and are provided with free text boxes to elaborate on their assessment. These free text comments are reproduced below.

ARTICLE DETAILS

Title (Provisional)

Study protocol for a randomised controlled trial to determine the efficacy of lisdexamfetamine for the treatment of acute methamphetamine withdrawal in inpatient settings

Authors

Acheson, Liam; Siefried, Krista J; Lintzeris, Nicholas; Dunlop, Adrian John; Haber, Paul S; Arunogiri, Shalini; Christmass, Michael; Doyle, Michael; Donoghoe, Mark; Nagle, Jack; Clifford, Brendan; McKetin, Rebecca; Lubman, Dan I; Brett, Jonathan.; Taylor, Nathan; Carr, Andrew; Levin, Frances R; Shoptaw, Steven; Ezard, Nadine

---

VERSION 1 - REVIEW

**Reviewer**	**1**
**Name**	**Tsui, Judith**
**Affiliation**	**University of Washington, Medicine**
**Date**	**25-Aug-2025**
**COI**	**None**

This is a protocol paper describing a double-blind, randomized, controlled trial to evaluate the efficacy of lisdexamfetamine in reducing symptom severity during acute methamphetamine withdrawal. The study will recruit 184 adults with mod-severe methamphetamine use disorder (MUD) who want withdrawal treatment and will randomize patients to receive either lisdeamfetamine taper or placebo for 5 days (250 mg day 1, reducing by 50 mg each day, then placebo “wash-out” on days 6 and 7) in an inpatient setting. The primary outcome is reduction in withdrawal symptoms over the 7 days as measured by the “Amphetamine Withdrawal Questionnaire.” Additional secondary outcomes are numerous (cravings, sleep, treatment satisfaction, depression, anxiety, cost-effectiveness, treatment utilization) and some are measured post-withdrawal treatment (Day 28 and 84). There is also an ancillary qualitative study to assess the experiences of Aboriginal and Torres Strait Islander participants. Strengths of the study are the rigorous study design and focus on a highly clinically relevant intervention. There are some weaknesses/issues that merit further edits/justification as follows:

1) Under “Trial Design” it states that the “primary endpoint of the study is the AWQ score at Day 7”, yet the statistical analysis section says that “repeated measures will be used to analyse the primary efficacy measure of the between-group difference in average AWQ score over 7 days from baseline.” If the primary outcome is an estimate of difference based on repeated daily measure would make this clearer from the beginning. Also, the following sentence that states “secondary endpoints are Days 28 and 84” provides insufficient detail.

2) There are a large number of “secondary” outcomes measured at different timepoints. Are any considered more exploratory?

3) In “Participants” section under eligibility exclusion criteria it states that participants are excluded if they defined as “high risk” on the C-SSRS screener—is there a specific threshold that can be named? Similarly, another exclusion is contradiction to LDX which includes “moderate to severe hypertension” but does not list specific cut-offs for blood pressure.

4) Under “Outcomes and measures” it lists a 28-day and 84-day outcome as differences in “self-reported MA use measure by the Timeline Followback method.” What is the specific measure that is being compared—is it number of days of use within the 28-day period?

5) A minor point, but within that same paragraph, the last sentence states that an outcome is “treatment utilization assessed four-weekly to Day 84.” It is a bit unclear what “four-weekly” means—it is every 4 weeks?

6) Under the “Intervention” paragraph it would be helpful to elaborate on the details of what “standard of care withdrawal management services” might consist of—what specific medications and psychosocial supports might be offered—and whether this was consistent at all sites. Similarly, more details are needed what “post-withdrawal” care might consist of and whether this would differ by site or patient subgroups.

7) The statistical analysis section seems somewhat underdeveloped (although I am not a biostatistician). The survival analysis description is unclear—what is the specific event that being used for the “time-to-event” analysis? Those outcomes that are proposed for survival analyses --“adherence to study drug” and “in-patient treatment” are not seemingly discrete events. Also, measuring over such a short period of time (7 days) does not seem to make sense. And conversely, why are discrete events like AE being considered as “categorical measures”?

8) How is “adherence” to study medication measured/defined? Are study medications administered and observed by clinical staff or are they self-administered? Can participants refrain from taking medications?

9) Related to the above, are there any “per-protocol” analyses planned?

10) In the discussion the authors appropriately identify a study weakness the requirement that participants have access to, and be admitted to, a hospital-based withdrawal management unit which limits generalizability and restricts participation from “people with caring responsibilities to participate.” Can the authors provide more details on the

context/setting of the study—specifically how common it is for these patients to access inpatient withdrawal management for MUD? Would there need to be policy/guideline shifts for this intervention to be widely adopted? Also, providing this treatment in hospital, inpatient setting does not only exclude those with caring responsibilities—there are many other reasons that patients with MUD may not find inpatient treatment an acceptable option (jobs, prior traumatic experiences, etc.).

11) Childhood ADHD is one of the baseline measures listed in Table 1. What is the rationale for measuring? Are there any subgroup analyses planned? One might wonder if lisdexamfetamine effects might differ among patients with ADHD.

12) Is this conceptualized/designed as a Phase 2 or 3 study? Readers may wonder.

13) The Spirit Checklist page numbers did not seem to line up with the manuscript. Should there have been a protocol included as supplemental? The checklist refers to "Protocol page 46" for example.

VERSION 1 - AUTHOR RESPONSE

Reviewer 1's comments	
Under "Trial Design" it states that the "primary endpoint of the study is the AWQ score at Day 7", yet the statistical analysis section says that "repeated measures will be used to analyse the primary efficacy measure of the between-group difference in average AWQ score over 7 days from baseline." If the primary outcome is an estimate of difference based on repeated daily measure would make this clearer from the beginning. Also, the following sentence that states "secondary endpoints are Days 28 and 84" provides insufficient detail.	Thank you for highlighting this omission. We have added detail early in the text to mention that AWQ scores will be collected daily during the in-patient period (Page 7), and have clarified that the outcome of interest is the average daily score while on-treatment. We have edited the description of the primary analysis in the statistics section: during further development of the Statistical Analysis Plan, we decided that it will not use a repeated-measures approach (Page 12-13)<br><br>We have edited the Trial Design section to say that secondary endpoints will be measured up to Days 7, 28 and 84 – further details of those endpoints are provided later in the text (Pages 7 and 9-10).
There are a large number of "secondary" outcomes measured at different timepoints. Are any considered more exploratory?	Thank you for noting this. We consider all these outcomes as secondary rather than exploratory.
In "Participants" section under eligibility exclusion criteria it states that participants are excluded if they defined as "high risk" on the C-SSRS screener—is there a specific threshold that can be named? Similarly, another exclusion is contradiction to LDX which includes "moderate to severe	Thank you for noting this. We have added the requirements for high risk by the CSSRS-S into the methods and clarified the hypertension cutoffs based on the Australian Hear Foundations guidelines at systolic blood pressure ≥ 160mmHg and/or diastolic BP ≥ 100 mmHg (Page 8).

hypertension" but does not list specific cut-offs for blood pressure.	
Under "Outcomes and measures" it lists a 28-day and 84-day outcome as differences in "self-reported MA use measure by the Timeline Followback method." What is the specific measure that is being compared—is it number of days of use within the 28-day period?	Yes, that is correct. We have revised to clarify the TLFB outcomes (Page 9-10).
A minor point, but within that same paragraph, the last sentence states that an outcome is "treatment utilization assessed four-weekly to Day 84." It is a bit unclear what "four-weekly" means—it is every 4 weeks?	Yes – we have amended to "every four weeks" for clarity (Page 10)
Under the "Intervention" paragraph it would be helpful to elaborate on the details of what "standard of care withdrawal management services" might consist of—what specific medications and psychosocial supports might be offered—and whether this was consistent at all sites. Similarly, more details are needed what "post-withdrawal" care might consist of and whether this would differ by site or patient subgroups.	Thank you – we have added examples of what the support may be at Page 10, however support will differ between site and individual patient needs so more detail cannot be added. Post-withdrawal care will be dictated by the patient's own goals and discharge plan, for example while one patient might go on to residential rehabilitation another might seek private counselling while working. Due to the highly individual nature of this no additional detail can be given.
The statistical analysis section seems somewhat underdeveloped (although I am not a biostatistician). The survival analysis description is unclear—what is the specific event that being used for the "time-to-event" analysis? Those outcomes that are proposed for survival analyses --"adherence to study drug" and "in-patient treatment" are not seemingly discrete events. Also, measuring over such a short period of time (7 days) does not seem to make sense. And conversely, why are discrete events like AE being considered as "categorical measures"?	Thank you for these comments. We have updated the statistical analysis section to address these issues. Discontinuation was the event of interest in the survival analysis, however since the number of days on-treatment is a discrete outcome with no censoring, we have updated the analysis plan to use ordinal logistic regression instead. For AEs, "categorical" was being used as a synonym for "discrete". We have also made some additional edits to update some aspects that have changed slightly while the Statistical Analysis Plan has been developed. Please see Pages 12-13.
How is "adherence" to study medication measured/defined? Are study medications administered and observed by clinical staff or are they self-administered? Can participants refrain from taking medications?	Adherence is defined by the number of medication doses taken, divided by the number of possible doses during admission. Medications are administered and observed by staff, but participants can refuse medication. This has been clarified in Pages 12-13
Related to the above, are there any "per-protocol" analyses planned?	Thank you for clarifying, no there are no additional per-protocol analyses planned
In the discussion the authors appropriately identify a study weakness the requirement that participants have access to, and be	Thank you for this insight. We agree that context is important and have added national treatment data regarding episodes of care for

admitted to, a hospital-based withdrawal management unit which limits generalizability and restricts participation from “people with caring responsibilities to participate.” Can the authors provide more details on the context/setting of the study—specifically how common it is for these patients to access inpatient withdrawal management for MUD? Would there need to be policy/guideline shifts for this intervention to be widely adopted? Also, providing this treatment in hospital, inpatient setting does not only exclude those with caring responsibilities—there are many other reasons that patients with MUD may not find inpatient treatment an acceptable option (jobs, prior traumatic experiences, etc.).	amphetamine withdrawal management as well as commented on agonist treatment structures already being in place for other drugs. We also agree that there are others that cannot access inpatient treatment, and have clarified this in Pages 15-16.
Childhood ADHD is one of the baseline measures listed in Table 1. What is the rationale for measuring? Are there any subgroup analyses planned? One might wonder if lisdexamfetamine effects might differ among patients with ADHD.	Childhood ADHD is being assessed as part of our demographic information to better describe the sample. This will allow future investigation into the impact that childhood ADHD has on treatment effectiveness with lisdexamfetamine
Is this conceptualized/designed as a Phase 2 or 3 study? Readers may wonder.	Conceptualised as a phase 3 but technically phase 4 as post-marketing of lisdexamfetamine
The Spirit Checklist page numbers did not seem to line up with the manuscript. Should there have been a protocol included as supplemental? The checklist refers to "Protocol page 46" for example.	Correct, the checklist refers to the protocol as it has greater detail than the protocol paper. Other papers in the journal do not necessarily upload the full protocol as supplementary however we are happy to defer to the editor’s decision on this
